# *In situ* observation of localized, sub-mm scale changes of phosphorus biogeochemistry in the rhizosphere

**DOI:** 10.1007/s11104-017-3542-0

**Published:** 2018-01-13

**Authors:** Andreas Kreuzeder, Jakob Santner, Vanessa Scharsching, Eva Oburger, Christoph Hoefer, Stephan Hann, Walter W. Wenzel

**Affiliations:** 1 Department of Forest and Soil Sciences, Institute of Soil Research, University of Natural Resources and Life Sciences, Vienna, Konrad-Lorenz-Strasse 24, A-3430 Tulln, Austria; 2 Land Salzburg, Natur- und Umweltschutz, Gewerbe (Abteilung 5), Michael-Pacher-Straße 36, A-5020 Salzburg, Austria; 3 Department of Crop Sciences, Division of Agronomy, University of Natural Resources and Life Sciences, Vienna, Konrad-Lorenz-Strasse 24, A-3430 Tulln, Austria; 4 Department of Microbiology and Ecosystem Science, Division of Terrestrial Ecosystem Research, University of Vienna, Althanstrasse 14, A-1090 Vienna, Austria; 5 Department of Chemistry, Vienna, University of Natural Resources and Life Sciences, Vienna, Muthgasse 18, A-1190 Vienna, Austria; 6 Austrian Centre of Industrial Biotechnology (ACIB), Muthgasse 18, 1190 Vienna, Austria

**Keywords:** Chemical imaging, Diffusive gradients in thin films, Planar optode, pH, Aluminium, Iron, Calcium, Magnesium, Manganese

## Abstract

**Aims:**

We imaged the sub-mm distribution of labile P and pH in the rhizosphere of three plant species to localize zones and hot spots of P depletion and accumulation along individual root axes and to relate our findings to nutrient acquisition / root exudation strategies in P-limited conditions at different soil pH, and to mobilization pattern of other elements (Al, Fe, Ca, Mg, Mn) in the rhizosphere.

**Methods:**

Sub-mm distributions of labile elemental patterns were sampled using diffusive gradients in thin films and analysed using laser ablation inductively coupled plasma mass spectrometry. pH images were taken using planar optodes.

**Results:**

We found distinct patterns of highly localized labile P depletion and accumulation reflecting the complex interaction of plant P acquisition strategies with soil pH, fertilizer treatment, root age, and elements (Al, Fe, Ca) that are involved in P biogeochemistry in soil. We show that the plants respond to P deficiency either by acidification or alkalization, depending on initial bulk soil pH and other factors of P solubility.

**Conclusions:**

P solubilization activities of roots are highly localized, typically around root apices, but may also extend towards the extension / root hair zone.

**Electronic supplementary material:**

The online version of this article (10.1007/s11104-017-3542-0) contains supplementary material, which is available to authorized users.

## Introduction

Plants actively modify their rhizosphere to render soil P plant-available by releasing protons, carboxylate anions and enzymes (Hinsinger and Gilkes 1995; López-Arredondo et al. 2014). Especially the root apices and younger root sections are highly active zones of P uptake (Colmer and Bloom 1998; Fang et al. 2007; Marschner et al. 2011), and also have been shown to be hot spots of organic anion exudation and proton release (Neumann and Römheld 1999; Raghothama and Karthikeyan 2005). High exudation rates of organic acid anions have been reported for dicots, especially for some *Fabaceae* species (Neumann and Römheld 1999). Some *Graminaceae* also show considerable exudation of carboxylates (Hinsinger 2001; Jones and Edwards 1998). As a side-effect of P solubilization by carboxylate anions and protons, mobilization of other elements like Al, Fe or Ca, which are not primarily targeted by the plant’s solubilization strategy, has been observed (Hinsinger 2001; Hinsinger and Gilkes 1995; Oburger et al. 2011). Even though Mn and P are not biogeochemically associated in soil, Mn is among the elements that are often co-solubilized with P. Lambers et al. (2015) pointed out that the common P solubilization mechanisms are also highly effective in solubilizing Mn, and therefore proposed shoot Mn as an indicator for plant P solubilization efficiency. As a consequence, the occurrence of Al, Fe and Ca, but also of Mn, in regions of elevated P concentrations in the vicinity of roots can serve as indicator of plant-mediated element solubilization.

Assessing the spatial distribution of root-induced changes of P solubility in the root zone is, however, experimentally challenging. While the formation of P depletion zones has been shown using rhizobox systems by slicing the soil in contact with the root mat, the resolution of this system is limited by the soil particle size if the rhizosphere layers are separated by nets or membranes (e.g. Youssef and Chino 1989). Refrigerated microtome sectioning can be used to obtain thinner slices of soil and thus better resolution of total P distribution, but the analysis of labile (extractable) forms of P is considerably biased by the cutting of soil particles, which exposes fresh mineral surfaces with differential solubility (Fitz et al. 2003). Moreover, the rhizobox approach only allows for the assessment of patterns perpendicular to root mats, which are composed of roots and root segments of different age (Wenzel et al. 2001), and thus represent ill-defined spatio-temporal averages of rhizosphere features. As an alternative, Wang et al. (2004) used micro-suction cups to obtain soil solutions at different distances from and locations along the root axis of rhizotron-grown plants (Göttlein et al. 1996). This approach allowed to monitor the temporal progress in the formation of depletion zones and related differences between the five studied plant species, but the spatial resolution was poor, distinguishing only zones of 0-1, 1-8, and >8 mm from the root plane. These and similar methods are obviously unable to assess the spatio-temporal heterogeneity of P availability and the impact of root activities on P depletion and mobilization along individual root axes.

An early method to study nutrient depletion along individual roots used autoradiography for visualizing isotopically exchangeable P depletion in ^33^P-labelled soil (Bhat and Nye 1973; Hendriks et al. 1981). However, techniques for specifically mapping the distribution and changes of labile, plant-available P around single, soil-grown plant roots at sub-mm scale became only recently available by combining ferrihydrite-impregnated diffusive gradients in thin films (DGT) hydrogels for P sampling with laser ablation inductively coupled plasma mass spectrometric analysis (LA-ICPMS) (Santner et al. 2010; Stockdale et al. 2008). In their study of the rhizospheres of two *Brassica napus* L. cultivars, Santner et al. (2012) not only imaged depletion zones of labile P at the sub-mm scale, but also found strongly increased labile P near root tips. Numerical simulations suggested highly localized P efflux from the roots, but could not rule out other sources such as P mobilization by localized exudation of organic anions or protons. While multi-analyte solute imaging was not available when the study of Santner et al. (2012) was conducted, the development of a novel DGT gel for simultaneous chemical imaging of anionic and cationic solutes now allows for the simultaneous localization of anions and cations (Kreuzeder et al. 2013). Moreover, the pH distribution can now be mapped before or after localized solute sampling using planar optodes (PO) (Blossfeld and Gansert 2007; Hoefer et al. 2017).

Using these tools, we conducted chemical imaging of labile P, Al, Fe, Ca, Mg, Mn and pH to further explore the biogeochemistry of P in the rhizosphere of three plant species with known, differential root activities and P acquisition strategies: (1) high release of organic acids from root apices (wheat), (2) strong rhizosphere acidification (buckwheat) and (3) high release of organic acids by proteoid roots (white lupine). The plants were grown on two P-limited soils with contrasting pH / carbonate content and were fertilized with different forms of nitrogen (NH_4_NO_3_, NH_4_^+^ and NO_3_^−^) to investigate the related root response, and its effects on labile P and associated elements along individual roots. The nitrogen treatments were used to induce variation in rhizosphere pH within a given plant-soil combination in order to create contrasting scenarios for the mobilization / immobilization of P and its associated elements. Compared to previous work, this combination of experimental design and novel imaging techniques was expected to allow for detailed localization of zones and hots pots of P depletion and accumulation along individual root axes, to relate the findings to different strategies of the plant species to cope with P limitation, and to explore the importance and patterns of P-associated elements in the rhizosphere in different soil – plant combinations.

Our hypotheses included:

1. Patterns of labile P are highly variable along individual root axes, typically showing P fluxes that are larger near root apices than in the bulk soil, and smaller towards basal parts.
2. The experimental plants are expected to modify their rhizosphere pH in the most active zones (root apex, extension zone) towards increasing P solubility.
3. Cationic elements that have either a role as P sorbents or as constituents of P minerals (Al, Fe, Ca) co-solubilize with P.
4. Increased fluxes of Mn can serve as indicators of root activities (proton and/or carboxylate release) for P mobilization and can therefore help to identify root-induced P mobilization zones.

## Materials and Methods

### Experimental Soils and Soil Analysis

Two experimental soils were used to cover different conditions for plant growth: carbonate-free soil (Gföhl) and a soil with high carbonate content (515 g kg^−1^; Lassee), both low in plant-available P concentration. The CAL P concentration class was A (very low) for the carbonate-free soil and B (low) for the calcareous soil (cf. CAL P content class A; see *Supplementary Information* (SI) document and Table 1). The unfertilized soils were collected in Lassee and Gföhl, Lower Austria. The soils were air dried and passed through a 2-mm sieve before use.

**Table 1:** General properties of the experimental soils

		Calcareous (Lassee)	Non-calcareous (Gföhl)
pH (H_2_O)		8.3	6.5
pH (CaCl_2_)		7.8	5.5
CaCO_3_ equivalent	g kg^−1^	515	0
	g kg^−1^	136	371
	g kg^−1^	547	406
	g kg^−1^	317	223
Soil textural class^a^		SiCL	L
Organic matter	g kg^−1^	51	15
P_ORG_	mg kg^−1^	206 (20)	452 (41)
P_CAL_^b^	mg kg^−1^	30.2	13.7
Al_AAO_^c^	g kg^−1^	0.683 (0.016)	1.05 (0.03)
Fe_AAO_^c^	g kg^−1^	1.51 (0.03)	2.79 (0.15)
Al_CBD_^d^	g kg^−1^	0.34 (0.25)	2.60 (0.15)
Fe_CBD_^d^	g kg^−1^	1.98 (0.58)	19.7 (0.3)
Al_AR_^e^	g kg^−1^	14.6 (0.4)	32.0 (1.9)
Ca_AR_^e^	g kg^−1^	222 (4)	8.34 (0.29)
Fe_AR_^e^	g kg^−1^	14.2 (0.2)	45.0 (1.3)
Mn_AR_^e^	g kg^−1^	0.30 (0.01)	1.31 (0.04)
P_AR_^e^	mg kg^−1^	717 (28)	603 (35)

^a^according to (FAO 2006), SiCL: Silty Clay Loam, L: Loam; ^b^ calcium acetate lactate extractable; ^c^ acid ammonium oxalate extractable; ^d^ citrate-dithionite-bicarbonate extractable; ^e^ *aqua regia* extractable; parenthesis show the standard deviation of three replicate measurements

Plant available P was determined using the calcium-acetate-lactate extraction (CAL) which is the Austrian standard for soil P testing (OENORM L1087 2012; Schüller 1969). Organic soil P was determined as difference between P extractable by 0.5 mol L^−1^ H_2_SO_4_ in combusted and uncombusted soil (Kuo 1996). Amorphous Fe and Al (oxy)hydroxides were determined by extraction of the soils with acid ammonium oxalate (AAO) at pH 3, total free Fe and dithionite-extractable Al using the citrate-bicarbonate-dithionite method (CBD), both according to Loeppert and Inskeep (1996). Total concentrations of Al, Fe, Ca and P were determined by *aqua regia* digestion (HCl: HNO_3_ = 3: 1; *v*/v) in a microwave-assisted digestion system (Multiwave 3000, Anton Paar, Graz, Austria). Results of the soil analysis are shown in Table 1.

All soils were fertilized with K, Mg, S and Zn (145 mg kg^−1^ KCl, 134 mg kg^−1^ MgCl_2_ × 6 H_2_O, 34 mg kg^−1^ ZnSO_4_ × 7 H_2_O) to ensure that plant growth was not limited by these nutrients. Additionally, three different N fertilization treatments were applied to induce pH effects in the rhizosphere: (1) 534 mg kg^−1^ Ca(NO_3_)_2_ × 4 H_2_O (expected response: alkalization); (2) 242 mg kg^−1^ NH_4_Cl (acidification), and (3) 181 mg kg^−1^ NH_4_NO_3_ (no pH change). Each treatment added 63 mg kg^−1^ N to the soil; the treatments are termed NO_3_^−^, NH_4_^−^ and NH_4_NO_3_-treatment throughout the manuscript. To prevent the nitrification of ammonium, a nitrification-inhibitor (45 mg kg^−1^ dicyandiamide) was added to all soils (Gahoonia et al. 1992). The fertilizers were prepared as salt solutions that were added to subsamples of the soil. After air drying, the subsamples were crushed and mixed thoroughly back into the remaining soil.

### Plant Experiments

The experimental plant species to investigate different plant P mobilization strategies were *Triticum aestivum* L. cv. Carazinho (wheat), *Fagopyrum esculentum* Moench (buckwheat) and *Lupinus albus* L. (lupine). The Carazinho wheat cultivar is known for its high release of organic acids mainly at the root apices (Delhaize et al. 1993; Ryan et al. 2014), buckwheat for its strong rhizosphere acidification (Hinsinger 2001) and lupine for its high release of organic acids by proteoid roots (Keerthisinghe et al. 1998; Neumann and Römheld 1999). Throughout the text, the plant species are referred to as W (wheat), B (buckwheat) and L (lupine).

Three different plant experiments were carried out: (1) A pot experiment for studying total carbon, citrate and malate exudation by wheat and buckwheat, with NO_3_^−^, NH_4_^+^ and zero-N fertilization treatments, and the two experimental soils. This experiment was carried out in triplicate, yielding 36 planted pots. (2) A set of preliminary chemical imaging runs with lupine, wheat and buckwheat as the experimental plants, NH_4_NO_3_ as N fertilization treatment, and the two experimental soils. For these experiments, which served for testing the experimental procedures, 2 plants were grown per treatment, with a total setup of 12 plant growth containers (rhizotrons, see below). Among other parameters, two DGT exposure times, 6 h and 24 h, were tested. As no principal differences in the DGT imaging results were observed, 6 h were chosen as exposure time for the main experiment. (3) A main chemical imaging experiment with wheat and buckwheat as the experimental plants, NO_3_^−^ and NH_4_^+^ fertilization treatments, and the two experimental soils. This experiment was carried out in triplicate, thus a total of 24 rhizotrons were set up.

All plant experiments were conducted in a plant growth laboratory with a controlled photoperiod of 16 h, a temperature of 25 – 30 °C and a photon flux density of 250 - 350 μmol m^−2^ s^−1^. Seedlings were germinated on wet tissue papers for 1-2 days (buckwheat), 3-4 days (wheat) or 1-3 days (lupine), subsequently, two seedlings were planted in close proximity to the front plate of the experimental rhizotrons (see below) or into the experimental pots.

### Plant Growth for Chemical Imaging

Plants were grown in perspex rhizotrons with detachable front plates (inner dimensions H × W × D = 40 cm × 10 cm × 1.5 cm). During the plant growth experiment, the transparent rhizotrons were covered with aluminium foil to prevent algal growth. Before filling the rhizotrons, the soil was moistened with a spray bottle filled with deionised water. The moist soil was gently tamped in layers to achieve a consistent bulk density of 1.0 – 1.1 kg L^−1^ (calcareous soil) and 1.2 - 1.3 kg L^−1^ (non-calcareous soil), based on soil dry weight. In the upper ~12 cm of the rhizotron the soil was filled into a separate compartment consisting of a plastic bag fitting the dimensions of the rhizotron. The weight of the rhizotrons was monitored daily and water was added to keep growth conditions constant. The upper soil compartment was kept moist at ~53% MWHC (maximum water holding capacity) for the whole experimental period while the lower compartment was kept dry until 21 – 23 DAG (days after germination). Root growth along the front side of the rhizotron was facilitated by tilting the rhizotrons at an inclination of 30°. After the initial growth period, after which the seed nutrient reserves were assumed to be depleted, the lower compartment was watered to ~53% MWHC through irrigation holes at the backside of the rhizotron and the plastic bag separating the two compartments was cut open to allow for root penetration into the lower soil compartment. The removable rhizotron lid was then exchanged with a perspex plate coated with a pH-sensitive planar optode film. When individual roots had grown at least 5 cm into the lower compartment, which occurred 2 – 5 days after opening the bag, PO pH measurements were performed. Immediately after pH imaging, the PO was removed and water was added to the lower compartment to achieve a water saturation of 75% MWHC. A clean front plate where the DGT gel was kept in place by a membrane (Nuclepore Track-Etched Membrane 0.2 μm, Whatman, UK) was applied for sampling labile P, Al, Fe, Ca, Mg and Mn. A schematic overview of this sampling setup is shown in Fig. S1 of the *Supplementary Information*.

### Planar Optode Imaging

pH-sensitive planar optode (PO) sensors were used for the imaging of pH distributions in the soil and rhizosphere (Larsen et al. 2011; Santner et al. 2015). Planar optodes are a fluorescent assay that uses reversible fluorophores immobilized in a thin layer of an analyte-permeable matrix, in this case a polyurethane-based hydrogel (Hydromed D4, Advan Source biomaterials, Massachusetts, US). This fluorophore-containing layer is coated onto a solid, transparent support material, in this study onto perspex-plates matching the dimensions of the rhizotron front plates (Hoefer et al. 2017). The analyte distribution is imaged by exciting the fluorophore using LED lamps and taking a photo of the fluorescence light using a digital single lens reflex (DSLR) camera. In our study, the soil was separated from the PO by a Nuclepore membrane to protect the soil and roots from physical damage during rhizotron opening, and an additional layer of 30-μm thick nylon membrane (03-30/18, SEFAR, Switzerland) for maintaining a homogeneous optical background signal.

The spatial resolution achieved for the PO images was ~ 60 μm × 60 μm (Santner et al. 2015). The acquired PO images were split into their red, green and blue color channels. The green color channel captures the response of the pH fluorophore 2′,7′-dichloro-5(6)-N-octadecyl-carboxamidofluorescein (DClFODA) that was used in this study, while the red channel records the emission of the Ziegelrot reference dye. The green/red color intensity ratio was used for image calibration (Hoefer et al. 2017).

### DGT Imaging

Chemical imaging of labile P, Al, Fe, Ca, Mg and Mn was conducted based on the diffusive gradients in thin films (DGT) technique, which utilizes hydrogels containing binding materials (*i.e.* resin gels) to sample anionic and cationic solutes in soils and sediments (Hoefer et al. 2015; Santner et al. 2012; Williams et al. 2014). DGT samples solutes by exposing the resin gel, covered by a thin diffusion layer, to soil. As the targeted ions diffuse through the diffusion layer and get bound to by the resin gel, a diffusive gradient towards the resin gel is established, which drives ion diffusion through the diffusive layer.

In this study, a novel, specialized resin gel containing a mixture of Zr-hydroxide for binding oxyanions, and suspended particulate reagent – iminodiacetic acid (SPR-IDA) for binding metal cations, was applied (Kreuzeder et al. 2013). The diffusion layer was a 10-μm-thick polycarbonate membrane (Nuclepore, Whatman, 0.2 μm pore size). Very thin diffusion layers are generally used in chemical imaging DGT applications to minimize potential image blurring by lateral diffusion. After DGT sampling, laser ablation inductively coupled plasma mass spectrometry (LA-ICPMS) was used for analyzing the resin gels at a spatial resolution of 57-103 μm along the x axis, depending on the analytical settings, and 300 μm along the y axis, which was chosen as the inter-line distance during the laser ablation process.

Although DGT measurements can be interpreted as time-integrated porewater concentrations (*c*_DGT_, Zhang et al. 1995), their interpretation as solute fluxes (*f*_DGT_) towards the resin gel, calculated as

1 $$ {f}_{DGT}=\frac{M}{At}, $$

where *M*/*A* is the mass of analyte bound per unit gel area, which is the result obtained from LA-ICPMS analysis, and *t* is the gel application time, is more appropriate for interpreting chemical images (Santner et al. 2015). Therefore all calibrated DGT images in this work are shown as fluxes towards the DGT gel in pg cm^−2^ s^−1^.

Further application and analysis details for both methods, PO and DGT, are given in the *Supporting Information*.

### Image Processing and Evaluation

LA-ICPMS data processing was done with common spreadsheet software. Image processing was done using the free software ImageJ, downloadable at https://imagej.net. Profile plots were generated using Systat Sigmaplot. Figure arrangement was done with Adobe Photoshop and Adobe Indesign.

As the first image evaluation step, the chemical images were evaluated for changes of the pH and of element fluxes in the rhizosphere by visual inspection of the chemical images and the corresponding photographs of the rooted soil area under investigation. If no visible changes of fluxes or pH relative to the corresponding background signal levels were observed, the plant specimen/element under evaluation was assigned as ‘no effect’. If changes of pH or elemental fluxes were visible, average pH values and/or elemental fluxes were calculated for ~1 mm^2^ areas in the respective rhizosphere segment and the bulk soil, respectively. pH changes and elemental flux increases or decreases were assigned, if one of the following criteria was met:

pH changes were classified as alkalization or acidification, if the average rhizosphere pH value differed from the average bulk soil pH by ≥0.2 pH units. Element flux increases were assigned, if the ratio of the rhizosphere segment showing the flux increase to the bulk soil flux was ≥1.3. Elemental flux increase features were distinguished into approximately circle-shaped features with diameters of up to ~3 mm, located at the position of root apices, and elongated zones of elemental flux increase along root axes that were up to ~10 mm long.

Areas of elemental flux decreases were classified as such if the ratio of the flux decrease to the bulk soil flux was <0.6. It is a specificity of the DGT technique, that features resembling element flux decreases in the rhizosphere can be generated by roots acting as diffusion barriers between soil and DGT gel: The gel area directly in contact with the root under investigation receives a very low solute flux compared to the adjacent soil, as the root is a physical barrier to diffusion. Thereby, thin root-shaped features can be generated that resemble element flux decreases. To avoid over-interpreting these features, element flux decrease zones with a width < 0.5 mm were classified as ‘element flux decrease not clearly extending beyond the root diameter’. For pH changes and elemental flux increases, no such diffusion-based features resembling actual chemical changes can be generated.

### Plant Growth for Porewater and Exudate Sampling

A pot experiment was carried out using similar conditions as for the rhizotrons (experimental plants: B and W; NO_3_- and NH_4_-fertilization treatments; nitrification inhibitor; identical light and watering conditions). After germination, two plant seedlings were transferred into pots filled with 170 g of soil, the same amount of soil as in the upper compartment of the rhizotrons. Rhizon porewater samplers (Rhizosphere Research Products B.V., Wageningen, The Netherlands) were installed in the pots to sample porewater from rooted soil at the end of the growth period (21 DAG for B and 23 DAG for W) for pH measurements.

Root exudates were sampled hydroponically 22 - 24 DAG, 2 h after the onset of light based on the water-immersion method described in Oburger et al. (2014). To this end, the soil was carefully washed off the plant roots by gentle rinsing with water until the roots were visibly clean. The roots were then further cleaned by submerging the plants in ~200 mL of deionized water containing 0.01 g L^−1^ Micropur classic (Katadyn ®), a silver-based biocide. After this cleaning procedure the roots were placed in 50 mL of sampling solution (deionized water containing 0.01 g L^−1^ Micropur classic) for 5 h in containers wrapped in aluminium foil to protect the sampling solution and roots from light. The sampling solution was syringe-filtered (0.45 μm, Nylon, Whatman; GE Healthcare, Freiburg, Germany), and measured for its dissolved organic carbon (DOC) and carboxylate anion concentrations. DOC was determined on a Vario TOC instrument (Elementar, Hanau, Germany).

### Carboxylate Anion Analysis

For carboxylate anion analysis, 100 μL of a uniformly labeled ^13^C yeast cell extract (Neubauer et al. 2012) were added to 10-25 mL subsamples of the exudate collection solutions as internal standard and samples were immediately frozen (−20 °C). Subsequently, the samples were lyophilized (−55 °C, 0.3 mbar; Beta 1-8 LDplus, Christ Gefriertrocknungsanlagen GmbH, Germany) and resuspended in 1 mL of water for analysis on a GC-EI-TOFMS (Agilent 7890B gas chromatograph in combination with an Agilent 7200 GC-QTOFMS system). Prior to analysis, samples were dried again and subjected to a two-step derivatization performed online on a GERSTEL DualRail MultiPurposeSampler (MPSII, GERSTEL, Germany). Derivatization as well as the GC temperature gradient was applied as described in Chu et al. (2015). For ionization, the ion source temperature and emission current applied to the filament were set to 230 °C and 35 μA, respectively. Absolute quantification of succinate, fumarate, malate, citrate and gluconic acid was done extracting the following accurate fragment masses: 173.0628, 217.0711, 335.1161, 465.1611, 333.1368 Th with a mass extraction window of 30 ppm. For internal standardization a uniformly ^13^C labeled standard was employed. The following fragment masses were extracted for the respective analytes: 177.0763, 220.0813, 339.1295, 471.1812, 337.1502 Th.

### Statistical Analysis

Analysis of variance (ANOVA) with the Student-Newman-Keuls test as a post-hoc test was used for identifying significant differences in the pH and exudate data at *P* ≤ 0.05.

## Results

### Pot Experiment for Exudate Sampling

**Fig. 1:**
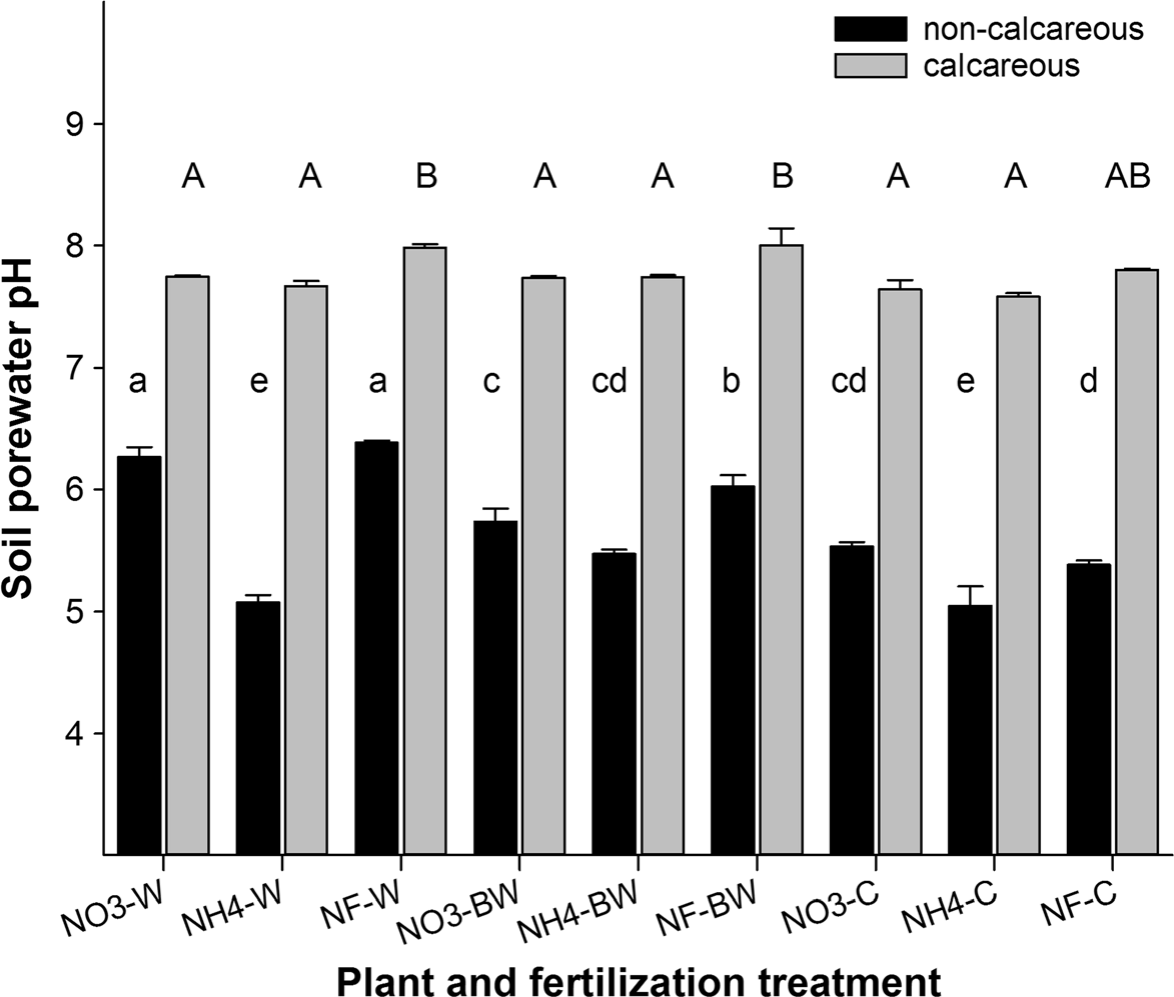
pH values measured in soil porewater obtained using Rhizon samplers. Letters indicate significant differences within the non-calcareous soil (small letters) and the calcareous soil (capital letters) (*P* ≤ 0.05). Error bars indicate the standard error (*n* = 3)

Soil porewater pH (Fig. 1) is in good agreement with the soil pH values measured in 0.01 mol L^−1^ CaCl_2_ and H_2_O as reported in Table 1. The differences among plant and fertilizer treatments in the calcareous soil were partly significant (P ≤ 0.05), but marginal (<0.3 pH units). In the non-calcareous soil, soil porewater pH tended to increase in the planted pots compared to the non-planted controls. This effect was less pronounced in the fertilized treatments, especially if nitrogen was applied in the form of ammonium, compared to the unfertilized treatment.

Total carbon release rates from B roots (Fig. S2 in the *Supporting Information*) were significantly less (9 – 13 nmol C g^−1^ root dwt. s^−1^) than from W roots (11 - 34 nmol C g^−1^ root dwt. s^−1^) (P ≤ 0.05). The differences within the carbon exudation rates of B were small and not significant (P ≤ 0.05). Wheat tended to release about two times more carbon into the NO_3_-fertilized, non-calcareous soil, however this difference was not significant. For W grown on the calcareous soil, the total C exudation rates were more than doubled and statistically significant (P ≤ 0.05) if fertilized with ammonium as compared to the non-fertilized control, while a smaller and insignificant increase was found if nitrate was used as nitrogen source.

The release of malate and citrate by roots is shown as the root dry weight-normalized exudation rate (Fig. S2c, S2d, *Supporting Information*). As the variation between replicates was rather large, only few statistically significant differences (*P* < 0.05) could be confirmed: NH_4_-fertilized W and B on calcareous soil exuded significantly more citrate than unfertilized W and NH_4_- and NO_3_-fertilized B on non-calcareous soil. Moreover, there was a non-significant trend for B grown on non-calcareous soil to exude smaller amounts of malate upon fertilization.

### Chemical Imaging of Labile P, Al, Fe, Ca, Mg, Mn and pH in the Rhizosphere

**Fig. 2:**
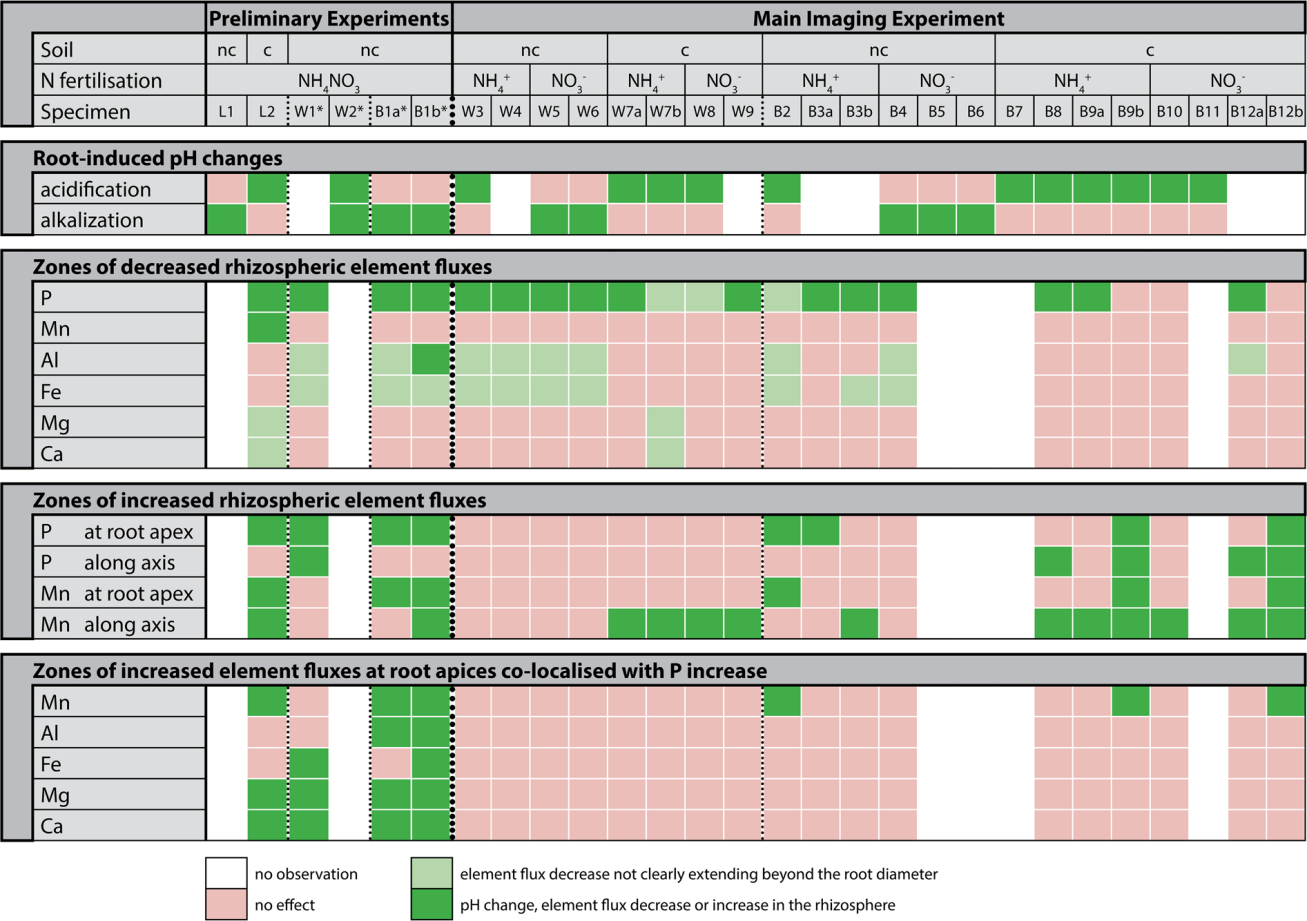
Overview on plant-induced changes in the rhizosphere. ‘L’: lupine; ‘W’: wheat; ‘B’: buckwheat. Columns between dashed lines contain data for the same plant species. ‘a’ and ‘b’ denote chemical images from different locations of one root system. ‘nc’ denotes non-calcareous soil, ‘c’ denotes calcareous soil. * denotes DGT deployment times of 24 h instead of the standard 6 h. Classification details for decreased element flux patterns not clearly extending beyond the root diameter (denoted in light green) and extending into the rhizosphere (denoted in dark green) is given in the M&M section

Due to insufficient rooting of the lower soil compartment, air bubble formation during the DGT or PO application and other sampling-related problems, chemical imaging could only be carried out successfully in 5 out of 12 rhizotrons in the preliminary experimental runs and in 18 out of 24 rhizotrons for the main chemical imaging experiment. In 5 of the 23 rhizotrons with successful imaging results, two instead of one DGT gels were applied simultaneously to different root segments. Therefore, we obtained 28 datasets with either DGT and PO data, or only DGT or PO data being available (Fig. 2).

The main effects of root activities on pH and element fluxes in the rhizosphere soil, as compared to the surrounding bulk soil, are:

1. The most obvious pattern relates to the root-induced changes of the rhizosphere pH in the two experimental soils. In the calcareous soil, plant roots strongly and consistently acidified the rhizosphere across all plant and fertilizer treatments. In contrast, the rhizosphere was consistently alkalized in the carbonate-free soil, except of the NH_4_^+^ treatments where we observed acidification.
2. Zones of decreased P fluxes were almost consistently found across both experiments and all treatments. These zones extended into the rhizosphere in 16, did not clearly extend beyond the root diameter in 3, and were absent only in 3 out of 22 DGT image datasets.
3. Zones of decreased Al and Fe fluxes not clearly extending beyond the root diameter were only - yet almost consistently - observed in the carbonate-free soil along B and W roots, independent of the form of nitrogen (NH_4_^+^, NO_3_^−^ or NH_4_NO_3_) applied. They were generally associated with alkalization, except in two NH_4_^+^ treatments with B and W. The only observation of decreased Al fluxes clearly extending into the rhizosphere was found for B on non-calcareous, NH_4_NO_3_-treated soil (specimen B1b).
4. Zones of decreased Ca and Mg fluxes were observed only in two DGT image datasets of the calcareous soil with (a) NH_4_NO_3_-fertilized L, and (b) NH_4_^+^-fertilized W, both after 6 h DGT deployment. As for the decreases observed for Al and Fe, those zones did not clearly extend beyond the root diameter.
5. In the main experiment, increased P fluxes along the root axis were observed in four out of six DGT image datasets of the B rhizosphere on calcareous soil, independent of fertilizer treatment (NH_4_^+^ or NO_3_^−^). A similar observation was made only once in the preliminary experiment for W grown on the carbonate-free soil with NH_4_NO_3_ fertilization.
6. In the preliminary experiment we found increased P fluxes at root apices, independent of soil and plant treatment. This observation was consistently associated with co-localized, increased fluxes of Ca and Mg, and in three out of four images with increased Mn fluxes. Increased, co-localized Al and Fe fluxes were only observed in two out of four DGT datasets. Increased P fluxes at root apices were also observed in the main experiment but were limited to 50% of the images with B as experimental plant, however, independent of soil and fertilizer treatment. In three DGT image datasets the increased P fluxes were co-localized with increased labile Mn.
7. Zones of increased Mn fluxes along root axes without concurrent increase of labile P or other elements were almost consistently observed for W grown on the calcareous soil, being more pronounced with NH_4_^+^ as compared to NO_3_^−^ fertilization. Similar, but less developed zones of increased labile Mn without increase of other element fluxes also occurred in 3 DGT image datasets of B grown on either soil. Increased Mn fluxes in the absence of the mobilization of other elements were consistently associated with rhizosphere acidification.
8. Zones of concomitantly increased Mn and P fluxes along root axes were identified only for B on the calcareous soil, two times each in the NH_4_^+^ and NO_3_^−^ treatments, respectively. Again, this was consistently associated with rhizosphere acidification.
9. Zones of strongly decreased labile P along root axes with concurrent increase of labile P at the root apex were consistently found in the preliminary experiment, independent of the soil and plant treatment, but were limited to NH_4_^+^-fertilized B grown on the calcareous soil in the main experiment. The observations were not related to any particular pattern of pH change in the rhizosphere.
10. Decreased Mn fluxes in the rhizosphere were observed only in one DGT image dataset on the calcareous soil planted with L in the preliminary experiment.

**Fig. 3:**
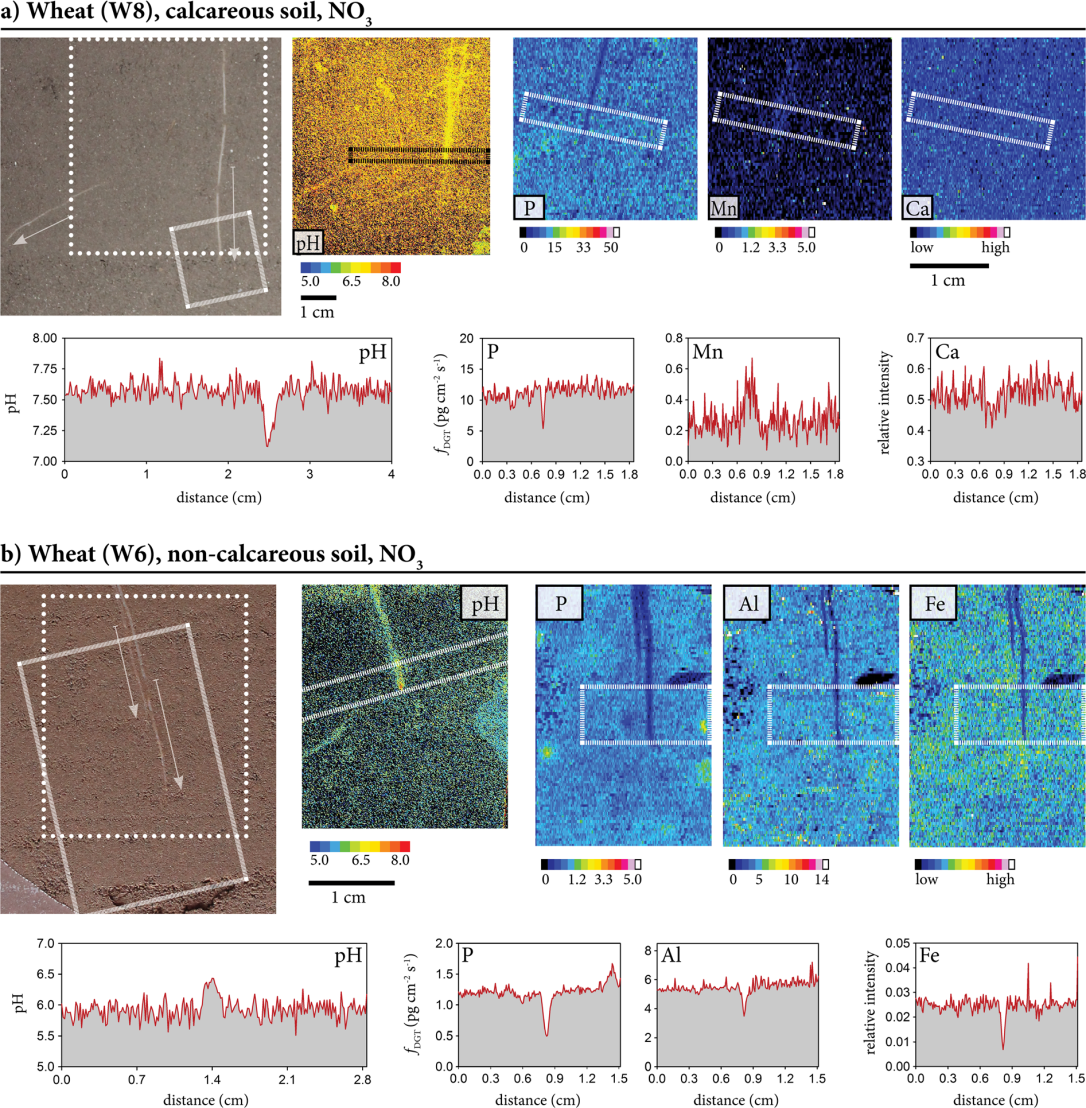
Characteristic patterns of labile element fluxes and pH in the rhizosphere. **(a)** Wheat grown on calcareous soil with NO_3_ fertilization, (**b**) wheat grown on non-calcareous soil with NO_3_ fertilization. Each image set contains a photograph of the root with arrows indicating root growth between the application times of the PO and DGT. Scale bars represent 1 cm; the smaller scale bar in pane (a) corresponds to the photo and the pH image, the larger one to the element images. The imaging areas for the PO are indicated with dotted lines in the photos, the imaging areas for DGT are indicated with dashed lines. Profiles show pH and elemental flux (pg cm^−2^ s^−1^). The long, rectangular boxes in the chemical images indicate the area used to calculate the profile plots by vertically averaging the single-pixel DGT fluxes. PO images were noise corrected (black pixels represent image noise where no pH-value could be assigned)

**Fig. 4:**
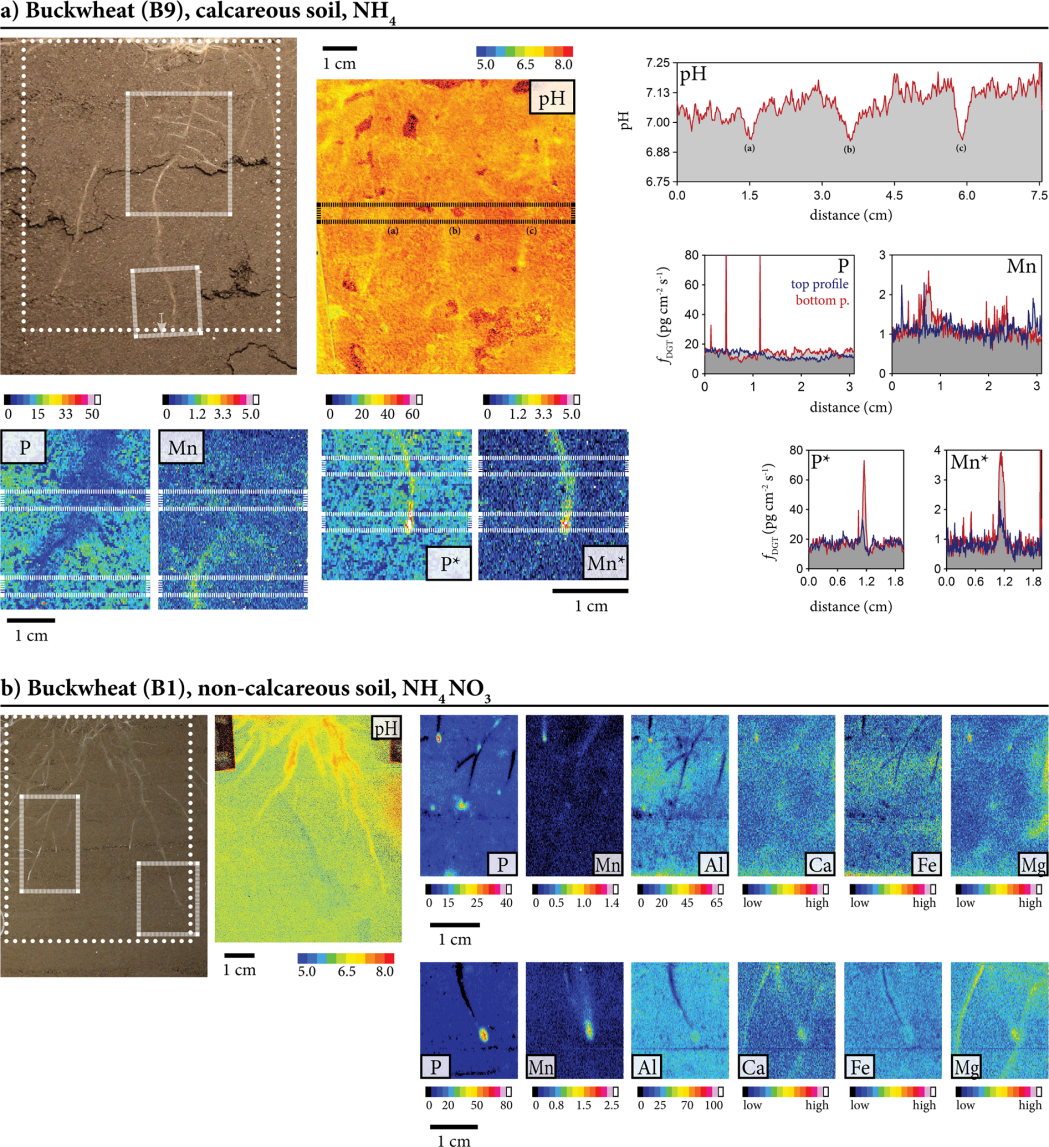
Characteristic patterns of labile element fluxes and pH in the rhizosphere. (**a**) Buckwheat grown on calcareous soil with NH_4_ fertilization, (**b**) buckwheat grown on non-calcareous soil with NH_4_NO_3_ fertilization. Scale bars represent 1 cm. In each pane, individual scale bars correspond to the photo and the pH image, and to the two different DGT images. The imaging areas for the PO are indicated with dotted lines in the photos, the imaging areas for DGT are indicated with dashed lines. Profiles show pH and elemental flux (pg cm^−2^ s^−1^). The long, rectangular boxes in the chemical images indicate the area used to calculate the profile plots by vertically averaging the single-pixel DGT fluxes. PO images were noise corrected (black pixels represent image noise where no pH-value could be assigned)

**Fig. 5:**
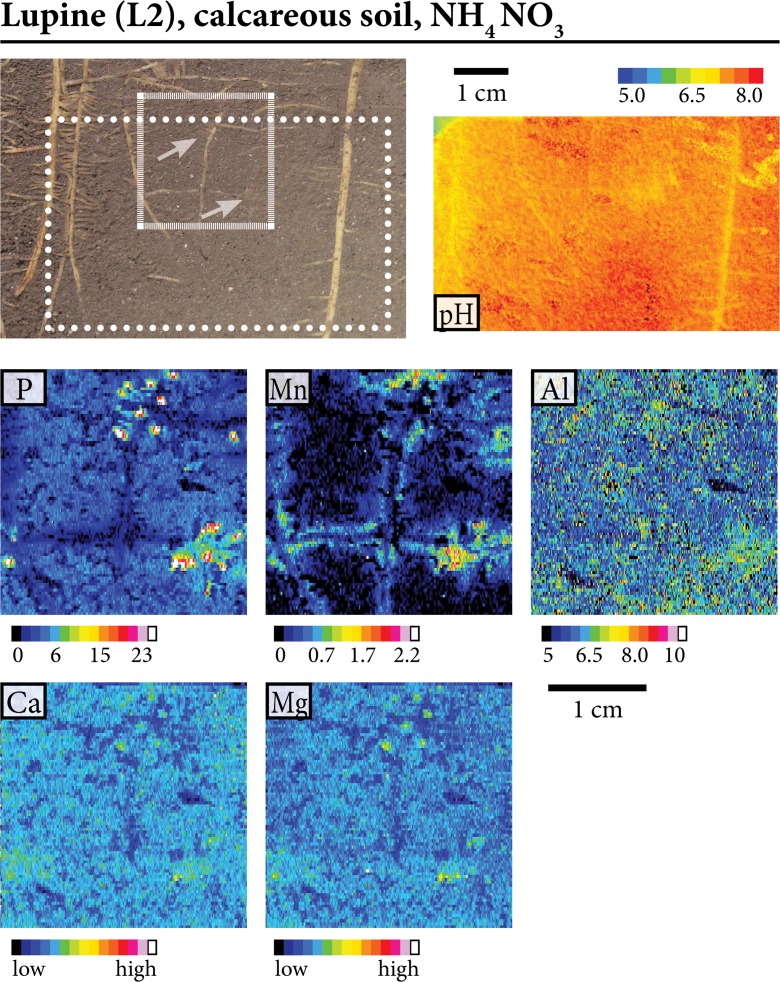
Characteristic patterns of labile element fluxes and pH in the rhizosphere of lupine grown on calcareous soil with NH_4_NO_3_ fertilization. Scale bars represent 1 cm. The smaller scale bar corresponds to the photo and the pH image, the larger one to the element images. The imaging areas for the PO are indicated with dotted lines in the photos, the imaging areas for DGT are indicated with dashed lines. PO images were noise corrected (black pixels represent image noise where no pH-value could be assigned)

In the following, some of the described patterns are exemplified in the chemical images shown in Figs. 3, 4 and 5 and Fig. S3 (*Supporting Information*). Figures 3a, b show root segments of NO_3_^−^-fertilized W grown in calcareous (3a, corresponding to W8 in Fig. 2) and carbonate-free soil (3b, W6 in Fig. 2), clearly demonstrating the general pattern of opposite direction of pH change in the two experimental soils, with acidification in the presence of carbonates and high soil pH (pH >7-8) versus alkalization in the moderately acidic (~pH 5-6), carbonate-free soil. The rhizosphere acidification in the calcareous soil is associated with decreased labile P not clearly extending beyond the root diameter, and increased Mn fluxes along the W root axis. Alkalization in the non-calcareous soil is accompanied by decreased fluxes of P, Al and Fe, the latter two not clearly extending beyond the root diameter.

Figure 4a shows root sections of B, including an older root segment with root branching and lateral roots (chemical images at upper right side, B9b in Fig. 2) and a younger root segment with the root apex and the extension / root hair zone (chemical images at lower right side, B9a in Fig. 2). Both zones were imaged for pH and element fluxes, showing decreased labile P along the older root axis but co-localization of strongly increased labile P and Mn at the root apex, expanding towards the extension / root-hair zone. The observed changes of P and Mn lability are associated with acidification (~0.3 pH units) along most of the root axis. Figure 4b shows the images of two terminal root axes of the root system of a single B specimen, corresponding to B1a and B1b in Fig. 2. Both root segments show decreased fluxes of P, Al and Fe not clearly extending beyond the root diameter in the older sections, and typically strongly increased labile P, Al, Fe, Ca, Mg and Mn at / near the root apex of NH_4_NO_3_-fertilized B grown in the carbonate-free soil. Figure 5 shows similar patterns for a root segment of L grown in the same soil and with the same fertilizer treatment. However, zones of decreased element fluxes were observed here for Ca and Mg, both not clearly extending beyond the root diameter, but not for Al and Fe (L2 in Fig. 2).

The temporal development of zones of decreased P fluxes in the rhizosphere of NH_4_^+^-fertilized wheat grown in the calcareous soil is demonstrated in Fig. S3 (*Supporting Information*). The right one of the two imaged neighboring root axes lags about 3 days behind the left root segment, resulting in less developed lateral extension of the zone of decreased P fluxes. Based on the images, an expansion rate of the P depletion zones of ~1 mm d^−1^, towards one side of the root, can be derived.

The zones of decreased labile P were found to expand up to 2.5 mm on each side from the root – soil interface. Generally, the width of these zones increases towards older root segments.

## Discussion

### Effect of DGT Sampling Time on Rhizosphere Element Patterns

Comparison of 24 h and 6 h DGT exposure times between the preliminary and main experiment revealed a clear effect of deployment time on element mobilization patterns, as zones of strongly increased fluxes of Al, Fe, Ca and Mg were only observed at root apices of W and B after the longer deployments in the preliminary experiment. In contrast, P and Mn mobilization was frequently observed after only 6 h of deployment in the main imaging experiment. Our findings indicate that P mobilization by plants can occur without co-solubilisation of other elements that are considered P solubility controls (e.g. Price 2006). Note that the longer deployment time required for Ca and Mg could also be related to their weaker binding to the DGT resin compared to (transition) metals like Fe and Al.

### Effect of pH changes and root exudation on labile P in the rhizosphere^1^

The pH measurements at the macroscale in soil porewater showed alkalization of the moderately acidic soil, without N-form-specific responses. The changes of pH observed in the chemical images at the microscale (see discussion below) in the rhizosphere of plants grown on the calcareous soil could not be captured by the pH measurements in the soil porewater at the macroscale, probably because of the strong buffering of the released protons by carbonates (Hinsinger 1998).

The organic compounds (DOC, malate, citrate) released from W and B roots obtained from the pot experiment do not allow for generalization of specific responses to soil or fertilizer treatments. However, the total C release rate is substantially larger from roots of W compared to B, which is in line with expectations from previous work (Possinger et al. 2013; Zhu et al. 2002). Given that P fluxes only increased in the acidified rhizosphere of B (and L) grown on the calcareous soil, we conclude that P mobilization in the B rhizosphere was linked to proton release rather than exudation of organic compounds.

Microscale chemical imaging of pH around roots grown in the acidic soil (pH_CaCl2_ 5.5) showed that the pH response varied with nitrogen form: ammonium-fertilized plants showed acidification, while the same amounts of N supplied as nitrate and ammonium nitrate resulted in alkalization. Both effects are in line with the known H^+^ release by roots upon NH_4_^+^ fertilization, as well as OH^−^ release following NO_3_^−^ uptake (Gahoonia et al. 1992; Riley and Barber 1971). The observed rhizosphere alkalization in the acidic soil in the absence of NH_4_^+^ fertilization could be a response to P deficiency in order to increase the phosphate concentration in soil solution. While we do not know the pH of minimal P solubility in our experimental soils, the relatively low P load suggests that it is likely at the lower end, *i.e.* around pH 4.5 - 5.5, of the reported minimum pH range (Barrow 2017; Eriksson et al. 2016). In terms of P load, pH and organic matter, the non-calcareous soil of our study is similar to soil Ekebo in the study of Eriksson et al. (2016), which has its minimum P solubility around pH 4.5. The observed pH increase from pH 5.5 (bulk soil, Fig. 4) to 6.5 near B root tips could therefore explain the observed strong increase in P fluxes after longer (24 h) deployment time. However, after shorter DGT deployment (6 h), alkalization consistently occurred without any changes in P solubility (Fig. 2).

In the calcareous soil (pH_CaCl2_ 7.8) chemical imaging revealed consistent acidification of the rhizosphere by up to 0.5 pH units, independent of plant species and the form of N supply (Fig. 2), thus masking the effect of OH^−^ ions released in response to NO_3_^−^ fertilization in this soil. For B and L this was often associated with increased P fluxes near the root apex and/or along the root axis while no P responses could be observed for W (Fig. 2). According to published minima of P solubility in soil (Barrow 2017; Eriksson et al. 2016; Weng et al. 2011) lowering pH from above 7.5 to about 7 should lead to decreased P solubility unless it is controlled by Ca-P minerals (Weng et al. 2011; Eriksson et al. 2016). In terms of P load, pH and organic matter, the calcareous soil of our study is comparable to soil Fors of Eriksson et al. (2016). P solubility in soil Fors showed a minimum at pH 7.8. With a bulk soil pH_CaCl2_ of 7.8, acidification of the calcareous soil in our study should result in P solubilization if Ca-P minerals are present, which is likely according to published information on P speciation in such high Ca soil (Eriksson et al. 2016; Luo et al. 2017; McLaren et al. 2015; Zhang et al. 2014), and in line with the measured increase of P solubility in soil Fors when pH was lowered from 7.8 to 7 (Eriksson et al. 2016).

### Spatial Pattern of Labile P

Zones of decreased P fluxes were observed along the root axis in all but 3 of 22 DGT image datasets. They are consistent with the commonly found rhizosphere P depletion, which is explained by the low solubility of P, the low effective diffusion coefficient of P in soil, and the high demand of plants (Hinsinger et al. 2005; Tinker and Nye 2000). As a consequence, uptake exceeds the rate of P resupply from the solid phase and by diffusion, leading to the formation of depletion zones (Hinsinger et al. 2005; Tinker and Nye 2000). Depletion zones of isotope-labelled P were imaged in previous work (e.g. Hendriks et al. 1981), but only recently high resolution chemical maps of labile P depletion in the rhizosphere of *B. napus* were obtained using DGT LA-ICPMS (Santner et al. 2012). The expansion of the depletion zones of labile P of up to 2-3 mm on each side of the roots (Figure S3) is well in line with that of modelled (Tinker and Nye 2000) and imaged isotope-labelled P depletion zones (Bhat and Nye 1973; Hendriks et al. 1981). Our results show that the findings of Santner et al. (2012) can be reproduced for different plant species, soil conditions and fertilizer treatments.

However, our imaging data reveal additional, complex spatial patterns of labile P in the rhizosphere. In addition to the depletion zones, we frequently identified zones of increased P fluxes at root apices, and in several images also expansion of increased labile P along the younger root axes, the latter almost exclusively found for B grown on the calcareous soil. Zones of P depletion in older root segments alternating with zones of increased fluxes at the root apex were typically found upon longer DGT deployment times (24 h) across all treatments, and in the rhizosphere of NH_4_^+^-fertilized B grown on the calcareous soil at shorter deployment (6 h). This pattern appears to be a general phenomenon but becomes more visible if the deployment of DGT is sufficiently long, or if strong acidification in younger root segments and root apices leads to substantial mobilization of P, as observed for B. Buckwheat is known for its ability to strongly acidify its rhizosphere (Possinger et al. 2013), which is likely to be further enforced by NH_4_^+^ fertilization (Gahoonia et al. 1992). Mobilization of P near root apices is consistent with observations of increased release of root products (protons, organic acid anions) at root tips and in the elongation zone (Blossfeld 2013; Hoffland et al. 1989; Ryan et al. 2014; Ryan et al. 2009).

However, zones of large fluxes of labile P at root apices also might be related to P release from plant roots as suggested by Santner et al. (2012). Here we show indication for – expected – mobilization of soil P at root apices but we cannot exclude P release by roots as additional P source. In one image (NH_4_^+^-fertilized B grown on non-calcareous soil, specimen B3a, Fig. 2), we even found strongly increased labile P at the root apex without any changes of the other elements´ lability. Further work is currently conducted in our laboratory to separate between the processes of root-induced P mobilization from soil constituents, and P release from root tissues.

Overall we show that plant roots mobilize or release P into the rhizosphere near their root apices, while the most active region for P uptake is behind the root apex, leading to formation of pronounced depletion zones in older root sections. The expansion of depletion zones appears to be fast (~1 mm d^−1^) as indicated by the comparison of images of root axes of known, differential age.

### Spatial Pattern of Elements Involved in P Biogeochemistry

Co-localized zones of increased P and its main solubility controls Al, Fe, Ca and Mg were consistently found at root apices in the preliminary experiment with 24 h DGT deployment. Given the relatively low signals in these images, it seems that a sampling period >6 h is needed for a sufficient amount of these elements to accumulate and be detectable on the DGT gels.

The observed coincidence of increased fluxes of P and the cationic elements Al, Fe, Ca and Mg in alkalized hot spots around root tips of B, grown in the acidic soil after 24 h DGT deployment, can only be partly explained by the observed alkalization. When increasing the pH from ~5.5 to 6.5, soluble Ca (and Mg) is expected to decrease while soluble Al and Fe are more likely to increase (Barrow 2017; Oburger et al. 2011; Weng et al. 2011). Therefore, and in line with the larger exudation rates measured for W as compared to B (Fig. S2), the observed element mobilization at the root tips of W is probably related to exudation of organic compounds (Neumann and Römheld 1999; Raghothama and Karthikeyan 2005).

In the calcareous soil, the observed increases in Ca and Mg, as well as co-localized increased P, were closely associated with the apices of two root clusters of lupine. These features were also co-localized with slight pH decreases. This release of Ca and Mg could be due to the dissolution of Ca- and Mg-carbonates and potentially also –phosphates, or by protonation of variable-charge surfaces and subsequent Ca and Mg desorption.

Independent of plant species and treatment, we also observed zones of co-localized, increased P and Mn fluxes at root apices after the longer deployment (24 h) in the preliminary experiment, and, only for B, also in the main experiment (6 h deployment). Moreover, concomitant increase of P and Mn fluxes were observed along the root axes of several B specimens grown in the calcareous soil. Increased labile Mn at root apices and along root axes were also frequently observed without co-localized increase in P or other element fluxes, and they were consistently associated with rhizosphere acidification. Consequently, it is likely that Mn is solubilized without a direct link to P mobilization. Our results are in line with Lambers et al. (2015) who suggested to use Mn concentrations in plant shoots as an indicator of a cultivar’s P mobilization efficiency as Mn is mobilized by the same root activities as P.

## Conclusions

Our findings reveal several reproducible patterns of the spatial distribution of labile P and its solubility controls at sub-mm scale. Based on detailed data analysis we conclude that the observed pH changes in the rhizosphere are related to P mobilization by plant roots, with either alkalization or acidification depending on initial bulk soil pH and other factors controlling P solubility.

We also show that root activities and related processes of mobilization are typically localized around root apices, but also expand towards the extension / root hair zone. Our novel technique of simultaneous chemical imaging of P and its solubility controls proved to be useful in identifying zones of P mobilization. Such emerging imaging tools for mapping pH and labile element distributions at the single-root scale can be used to identify localized mobilization or immobilization mechanisms.

## Electronic supplementary material

ESM 1 (PDF 722 kb)

## Footnotes

1 Labile P in the rhizosphere in relation to soil pH and P association with the soil solid phase is discussed here with reference to recent modelling and experimental results, which are briefly summarized in the *Supporting Information*.
