# Supplementary material for: In situ observation of localized, sub-mm scale changes of phosphorus biogeochemistry in the rhizosphere
Source: Plant Soil. 2018 Jan 13;424(1):573–89. doi: 10.1007/s11104-017-3542-0 (PMC5902520; doi:10.1007/s11104-017-3542-0)
Supplement: Supplementary file 1 — (PDF 722 kb) [file 11104_2017_3542_MOESM1_ESM.pdf]

Supporting Information

In situ observation of localized, sub-mm scale changes of phosphorus biogeochemistry in the rhizosphere

Andreas Kreuzeder[1,2], Jakob Santner[1,3], Vanessa Scharsching[1], Eva Oburger[1,4], Christoph Hoefer[1], Stephan Hann[5,6], Walter W. Wenzel[1]*

[1)] University of Natural Resources and Life Sciences, Vienna, Department of Forest and Soil Sciences, Institute of Soil Research, Konrad-Lorenz-Strasse 24, A-3430 Tulln, Austria

[2)] Land Salzburg, Natur- und Umweltschutz, Gewerbe (Abteilung 5), Michael-Pacher-Straße 36, A-5020 Salzburg, Austria

[3)] University of Natural Resources and Life Sciences, Vienna, Department of Crop Sciences, Division of Agronomy, Konrad-Lorenz-Strasse 24, A-3430 Tulln, Austria

[4)] University of Vienna, Department of Microbiology and Ecosystem Science, Division of Terrestrial Ecosystem Research, Althanstrasse 14, A-1090 Vienna, Austria

[5)] University of Natural Resources and Life Sciences, Vienna, Department of Chemistry, Vienna, Muthgasse 18, A-1190 Vienna, Austria

[6)] Austrian Centre of Industrial Biotechnology (ACIB), Muthgasse 18, 1190 Vienna, Austria

* To whom correspondence should be addressed. E-mail: jakob.santner@boku.ac.at

Plant P availability and soil pH. P availability to plants is generally considered to be controlled by Fe and Al in acidic soils, and by Ca at higher soil pH, therefore, plant P availability is commonly considered to be highest between pH 6 to 7 (Price, 2006). However, (Barrow, 2017) challenges this concept by presenting evidence for P availability to continuously increase as pH decreases from ~7 to ~4, as both P desorption from the solid phase and plant P uptake increase concomitantly with decreasing pH. Below pH 4, plant P uptake decreases again, which might be connected to increasingly soluble Al. In an experimental and modeling study on soil P desorption, Weng et al. (2011) observed, that P solubility remains low between pH 3 and 7 in low-P soils, whereas there was a solubility maximum around pH 4, low P solubility between pH 6-8, and an increase of P solubility between pH 8 and 10 in high-P soils. Eriksson et al. (2016) also reported experimental data on pH-dependent solubilization of P in non-calcareous and calcareous soils of Swedish long-term experiments. In the Swedish soils, a minimum of P solubility between pH 5 and 7 in unfertilized control soils was found, whereas no consistent pattern was observed in the fertilized treatments. This evidence suggests, that P solubility is generally low between pH 5 and 7, but the location of the minimum depends on P load and soil properties such as carbonate and organic matter content, the presence of polyvalent cations, and distinct P minerals.

Association of P with the soil solid phase. Recent advances in solid-state P speciation techniques such as XANES revealed Ca phosphate minerals (apatites) derived from the parent material mainly to be present during the earliest stages of soil formation. Apatites dissolve during weathering, and the contained P redistributes to organic forms and is sorbed to Fe and/or Al oxides and hydroxides (Liu et al., 2013; Prietzel et al., 2013). Therefore, native, low P soils typically contain no or only small amounts of Ca phosphates, especially in the presence of larger amounts of Fe (Eriksson et

al., 2016; Hashimoto and Watanabe, 2014; Zhang et al., 2014), while fertilized soils almost consistently show neo-formation of Ca phosphates in response to high P and Ca inputs (Eriksson et al., 2016; Luo et al., 2017; McLaren et al., 2015; Zhang et al., 2014). Ca phosphates can occur even in acidic, but continuously fertilized soils (Beauchemin et al., 2003). Moreover, defined Fe or Al phosphate minerals have been detected in soils, but these observations are scarce and typically show only minor amounts of these minerals (Beauchemin et al., 2003; McLaren et al., 2015). These detailed data on mineral P species in soils support the accepted concept of inorganic soil P being mainly associated with Fe and Al via sorption, and only small amounts being present as defined Ca, Fe and Al minerals.

Phosphorus content classes of the experimental soils. The P content classes used for P fertilization in Austria, based on calcium acetate lactate (CAL) extraction, are given in Table S1 (Baumgarten et al., 2017).

Table S1. Soil P status according to CAL P extraction.

P content class	Soil P status	CAL P content (mg/kg)
A	very low	below 26
B	low	26 – 46
C	sufficient	47 – 68
D	high	69 – 174
E	very high	above 174

Chemical Imaging Setup.

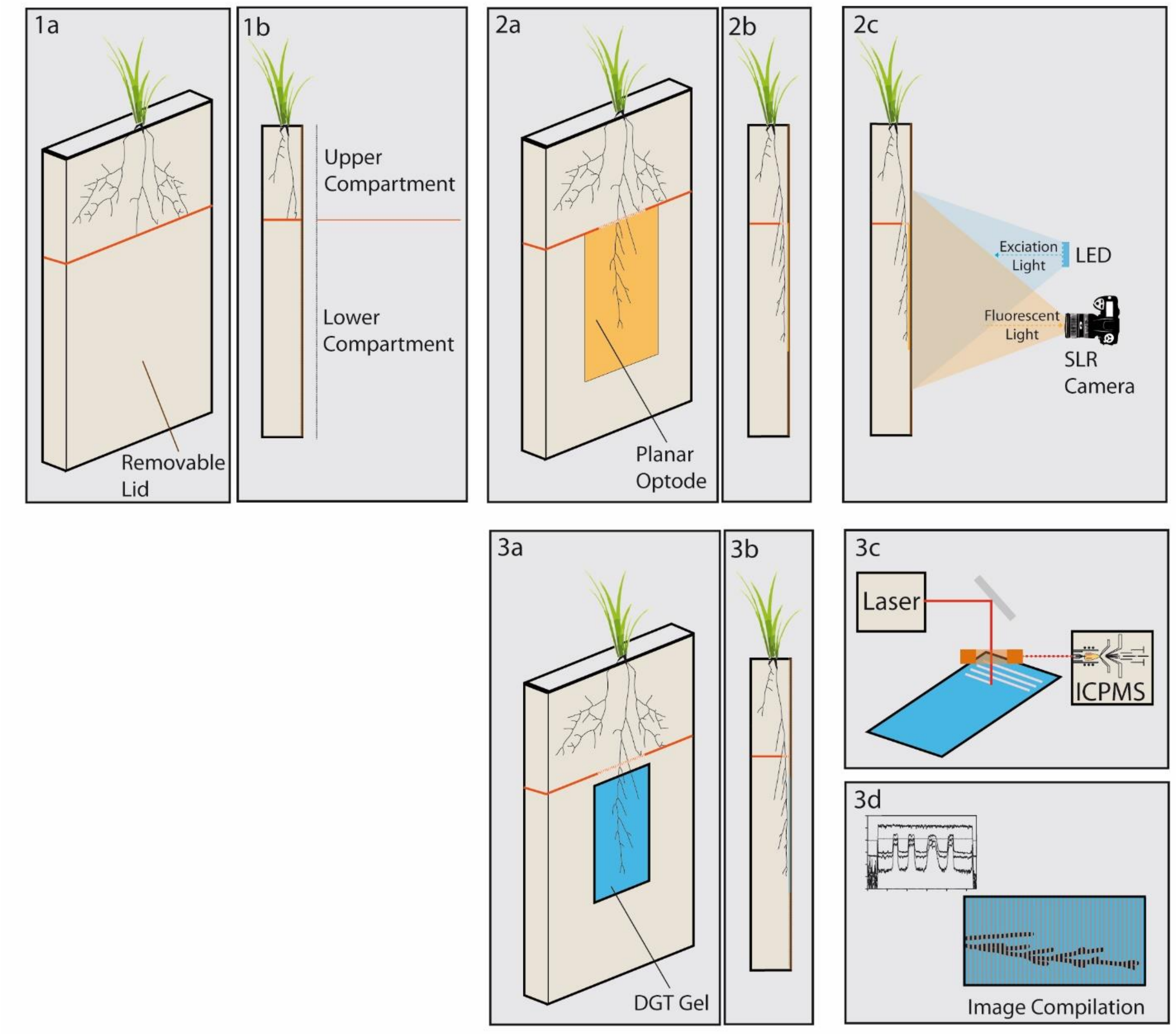

Figure S1. Overview on the sampling procedure of the chemical imaging experiment with (**1a**, **b**) the view and cross-section of the rhizotron with the upper and lower compartment, (**2a**, **b**) the application of the planar optode, (**2c**) the setup of the ratiometric pH measurement, (**3a**, **b**) the application of the DGT gel, (**3c**) the analysis of the dried gel by LA-ICP-MS and (**3d**) the image compilation from the scanned lines.

Optode sensor production. The sensing cocktail contained the lipophilic pH indicator DCIFODA (2´,7´-dichloro-5(6)-N-octadecyl-carboxamidofluorescein) and the reference dye Ziegelrot (Kremer Pigmente, Germany) (Hoefer et al., 2017), which were mixed at a 1 : 10 (w/w) ratio and dissolved in 14 % (w/w) polyurethane hydrogel (Hydromed D4, Advan Source biomaterials, Massachusetts, US) in a 3.5 : 1 (w/w) ethanol / HQ-water mixture. The cocktail was coated onto perspex plates as ~60-µm thick wet films using a knife-coating device. After solvent evaporation, this procedure yielded ~15 µm-thick optode sensing layers. Each PO was calibrated individually using in-house pH-buffers matched to the electrical conductivity of soil water extracts (non-calcareous soil: 407 µS cm^{-1}; calcareous soil: 1481 µS cm^{-1}) by adding NaCl. Sigmoid calibration curves were applied to the corresponding images for each optode individually.

DGT application and analysis. Gel sheets were produced and cut into pieces of approximately 6 × 4 cm. For the application the DGT gel sheets were put onto acid washed rhizotron front plates aligned with the place of interest for chemical imaging. This gel was cleaved to the front plate using a Nuclepore membrane which was fixed with strips of adhesive tape. The setup was then applied for the deployment period of 6 h / 24 h onto the rhizotrons. After retrieval, the DGT gels were put onto a polyethersulfone membrane (Supor 0.45 µm, Pall, USA) and dried in a gel dryer (Unigeldryer 3545, Laborgeräte and Vertriebs GmbH, Martinsried, Germany), which keeps the gels dimensionally stable. After drying, gel and membrane were inseparable from each other. The gel and membrane assembly was then mounted onto a glass plate using a double-sided adhesive tape. LA-ICP-MS analysis was performed on a quadrupole ICP-MS instrument (NexION 350, Perkin Elmer, USA) coupled to an excimer laser ablation system (UP 193-FX, ESI, NWR Division, CA, US).

The ablation system was set to 30 % energy output, 8 Hz laser pulse frequency, 100 µm laser spot diameter and 200 µm s^{-1} scan speed. Calibration standards were produced by spiking the gel solution with known amounts of the target analytes. As the upper layer of the HR-MBG gel is predominantly involved in the uptake of the analyte ions, analytes for calibration were only applied to the top gel layer. For the top layer a mixture of spiked aliquots of SPR-IDA (for Al, Ca, Mn) and Zr-Hydroxide/Hydromed D4 solution (for P) was used. This lead to a homogeneous loading of target analytes located at the top of the standard gels. Four standard gel sheets with varying loading were produced using this method and four gel discs were cut from each of these sheets. Three gel discs were digested using microwave assisted digestion while the fourth gel was used as laser ablation standard. The fourth gel disc replicate was dried and used as calibration standard during LA-ICP-MS (Kreuzeder et al., 2013). The isotopes ^{31}P, ^{27}Al, ^{44}Ca, ^{57}Fe, ^{55}Mn were analyzed along with ^{13}C, which was used as internal normalization standard.

Organic carbon, malate and citrate exudation of wheat and buckwheat.

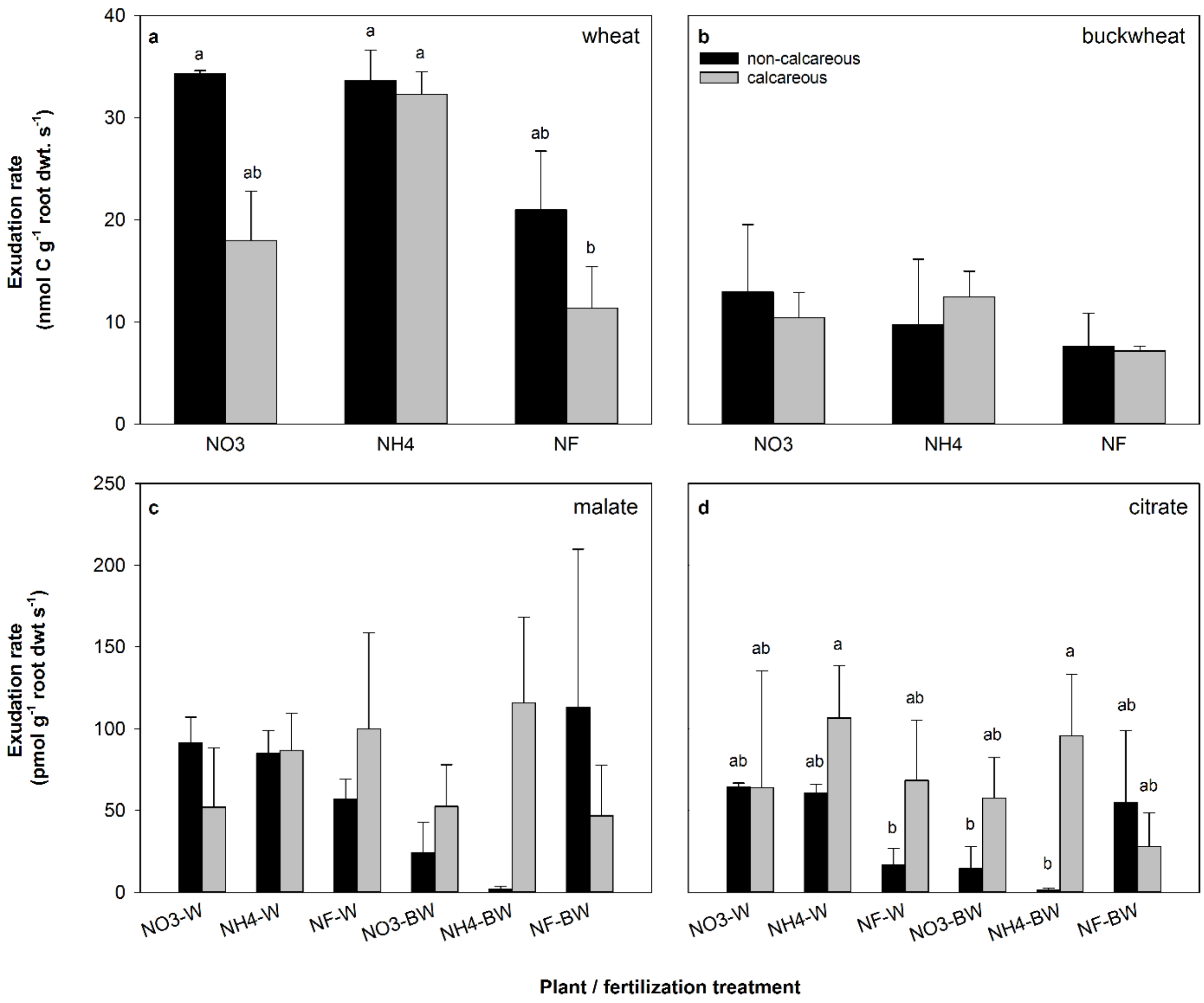

Figure S2. Release of organic carbon by **(a)** wheat and **(b)** buckwheat roots. **(c)** exudation of malate and of **(d)** citrate by wheat and buckwheat. NF: not fertilized. Error bars show the standard error (n = 3). Letters indicate significant differences between fertilizer×soil and fertilizer×plant×soil treatments (Student-Newman-Keuls, $P < 0.05$).

Development of P depletion zones around roots.

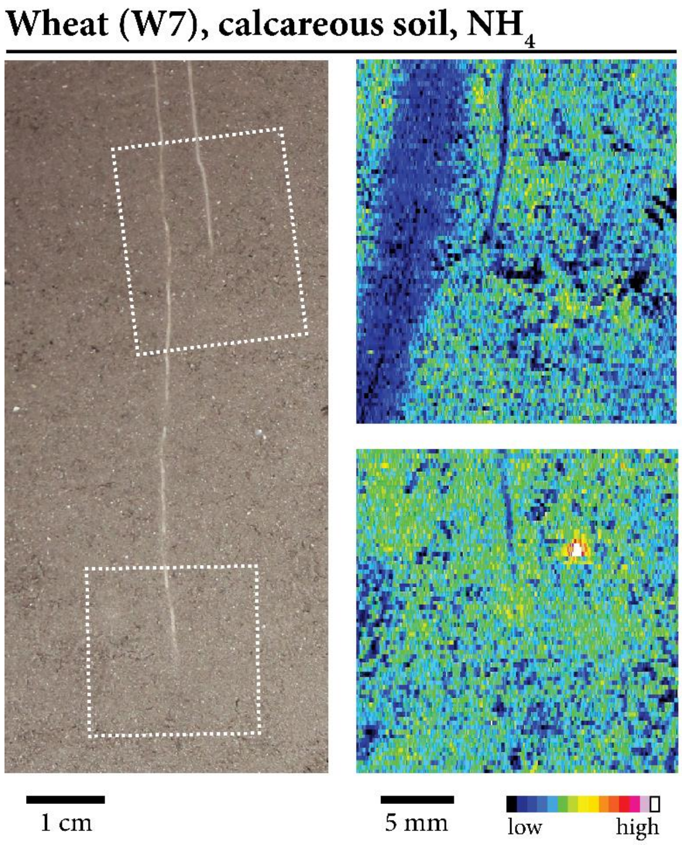

Figure S3. Development of a P depletion zone around a root of wheat. The shorter root (right root) had a delay of 3 days in comparison with the longer root. The imaging areas for the DGT are indicated with dotted lines. The relative P-flux is indicated in the calibration bar. The wheat plant was grown on calcareous soil with a NH_4 fertilizer treatment. The length of the scale bar is 1 cm.

Representativeness of this study and sampling related errors. DGT has been used as a sampling method for the investigation of sediments and soils previously and is a well-established technique (Lehto et al., 2012; Stockdale et al., 2008; Williams et al., 2014). In soils, however, sampling based on DGT requires a diffusive flux and therefore a relatively high water saturation is required which may lead to anoxia and changed redox-conditions. In this study, the used water saturation was kept as low as possible (50-80% of the maximum water holding capacity) to avoid such effects and the sampling was carried out swiftly. In previous work large Mn-patches were observed when high water saturation was used, which were related to redox-artefacts (Hoefer et al., 2015). In this study, no such effects were observed.

The occurrence of air bubbles or the behavior of plant roots sometimes impede the sampling with planar optodes and DGT gels. All experimental treatments were carried out in three replicates to ensure a complete dataset. The experimental data provided in Table 2 of the main document gives an overview on the observed plant variability and reproducibility of the results. Furthermore, the manipulation of the rhizotron covers always bears the possibility of root injury. This was avoided by using a protective membrane throughout the experiment and by gentle handling of the rhizotron covers. No obvious artifacts could be observed due to root injury in this study.

References

Barrow, N.J., 2017. The effects of pH on phosphate uptake from the soil. Plant Soil 410, 401-410.

Baumgarten, A., Berthold, H., Buchgraber, K., Dersch G., Egger, H., Egger, R., Eigner, H., Frank, P., Gerzabek, M., Hölzl, F. X., Holzner, H., Janko, M., Pernkopf, G., Peszt, W., Pfundtner, E., Pötsch, E. M., Rohrer, G., Schilling, C., Spanischberger, A., Spiegel, H., Springer, J., Strauss, P., Winkowitsch, C., Zethner, G., 2017. Richtlinie für die sachgerechte Düngung im Ackerbau und Grünland. 7th edition. https://www.ages.at/download/0/0/4bfee71413a6aa535d2e753fef27f17769bb2507/fileadmin/AGES2015/Service/Landwirtschaft/Boden_Datein/Broschueren/Richtlinien_fuer_die_sachgerechte_Duengung_im_Ackerbau_und_Gruenland_7_Auflage.pdf Last accessed 27.09.2017.

Beauchemin, S., Hesterberg, D., Chou, J., Beauchemin, M., Simard, R.R., Sayers, D.E., 2003. Speciation of phosphorus in phosphorus-enriched agricultural soils using X-ray absorption near-edge structure spectroscopy and chemical fractionation. Journal of Environmental Quality 32, 1809-1819.

Eriksson, A.K., Hesterberg, D., Klysubun, W., Gustafsson, J.P., 2016. Phosphorus dynamics in Swedish agricultural soils as influenced by fertilization and mineralogical properties: Insights gained from batch experiments and XANES spectroscopy. Sci. Total Environ. 566-567, 1410-1419.

Hashimoto, Y., Watanabe, Y., 2014. Combined applications of chemical fractionation, solution 31P-NMR and P K-edge XANES to determine phosphorus speciation in soils formed on serpentine landscapes. Geoderma 230-231, 143-150.

Hoefer, C., Santner, J., Borisov, S.M., Wenzel, W.W., Puschenreiter, M., 2017. Integrating chemical imaging of cationic trace metal solutes and pH into a single hydrogel layer. Anal. Chim. Acta 950, 88-97.

Hoefer, C., Santner, J., Puschenreiter, M., Wenzel, W.W., 2015. Localized Metal Solubilization in the Rhizosphere of Salix smithiana upon Sulfur Application. Environmental Science & Technology 49, 4522-4529.

Kreuzeder, A., Santner, J., Prohaska, T., Wenzel, W.W., 2013. Gel for simultaneous chemical imaging of anionic and cationic solutes using diffusive gradients in thin films. Analytical Chemistry 85, 12028-12036.

Lehto, N.J., Davison, W., Zhang, H., 2012. The use of ultra-thin diffusive gradients in thin-films (DGT) devices for the analysis of trace metal dynamics in soils and sediments: a measurement and modelling approach. Env Chem 9, 415-423.

Liu, J., Yang, J., Cade-Menun, B.J., Liang, X., Hu, Y., Liu, C.W., Zhao, Y., Li, L., Shi, J., 2013. Complementary phosphorus speciation in agricultural soils by sequential fractionation, solution 31P nuclear magnetic resonance, and phosphorus K-edge X-ray absorption near-edge structure spectroscopy. Journal of Environmental Quality 42, 1763-1770.

Luo, L., Ma, Y., Sanders, R.L., Xu, C., Li, J., Myneni, S.C.B., 2017. Phosphorus speciation and transformation in long-term fertilized soil: evidence from chemical fractionation and P K-edge XANES spectroscopy. Nutrient Cycling in Agroecosystems 107, 215-226.

McLaren, T.I., Guppy, C.N., Tighe, M.K., Schefe, C.R., Flavel, R.J., Cowie, B.C.C., Tadich, A., 2015. Validation of soil phosphate removal by alkaline and acidic reagents in a vertosol soil using XANES spectroscopy. Commun. Soil Sci. Plant Anal. 46, 1998-2017.

Price G. (Ed), 2006. Australian Soil Fertility Manual, 3rd Edition, Fertilizer Industry Federation of Australia and CSIRO. p 45.

Prietzel, J., Dümig, A., Wu, Y., Zhou, J., Klysubun, W., 2013. Synchrotron-based P K-edge XANES spectroscopy reveals rapid changes of phosphorus speciation in the topsoil of two glacier foreland chronosequences. Geochim. Cosmochim. Acta 108, 154-171.

Stockdale, A., Davison, W., Zhang, H., 2008. High-resolution two-dimensional quantitative analysis of phosphorus, vanadium and arsenic, and qualitative analysis of sulfide, in a freshwater sediment. Env Chem 5, 143-149.

Weng, L., Vega, F.A., Van Riemsdijk, W.H., 2011. Competitive and synergistic effects in pH dependent phosphate adsorption in soils: LCD modeling. Environ Sci Technol 45, 8420-8428.
Williams, P.N., Santner, J., Larsen, M., Lehto, N.J., Oburger, E., Wenzel, W., Glud, R.N., Davison, W., Zhang, H., 2014. Localized Flux Maxima of Arsenic, Lead, and Iron around Root Apices in Flooded Lowland Rice. Environmental Science & Technology 48, 8498-8506.
Zhang, M., Li, C., Li, Y.C., Harris, W.G., 2014. Phosphate minerals and solubility in native and agricultural calcareous soils. Geoderma 232-234, 164-171.
